# Die-off reaction of *Demodex* mites after treating demodicosis with oral ivermectin: A case report

**DOI:** 10.1016/j.jdcr.2024.07.006

**Published:** 2024-07-26

**Authors:** Anon Paichitrojjana, Anand Paichitrojjana

**Affiliations:** a School of Anti-Aging and Regenerative Medicine, Mae Fah Luang University, Bangkok, Thailand; b Faculty of Medicine, Ramathibodi Hospital, Mahidol University, Bangkok, Thailand

**Keywords:** *Demodex* mite, demodicosis, die-off reaction, ivermectin, rosacea

## Introduction

Demodicosis is a skin disorder caused by an abnormal increase in *Demodex* mites, common ectoparasites found in human pilosebaceous units. This condition can lead to various skin symptoms, including redness, dryness, follicular scales, papules, pustules, and roughness. It is also associated with specific conditions such as rosacea, perioral dermatitis, folliculitis, and blepharitis.^1^^,^^2^ The Standardized Skin Surface Biopsy (SSSB) is a method utilized to detect *Demodex* mites. This technique involves placing a 1 cm^2^ marked slide coated with cyanoacrylate glue on the affected cheek area for 1 minute, gently removing it, and then applying immersion oil for microscopic examination.

Diagnosis is based on suspected skin lesions confirmed by an abnormal density of *Demodex* mites with a SSSB >5 mites/cm^2^ and clinical improvement after acaricidal treatment.^2^ Several reports have documented the successful treatment of demodicosis and rosacea associated with *Demodex* mite infestation with oral ivermectin.^3^, ^4^, ^5^

We report a case of a patient with demodicosis who experienced a die-off reaction of *Demodex* mites following oral ivermectin treatment.

## Case report

**Fig 1:**
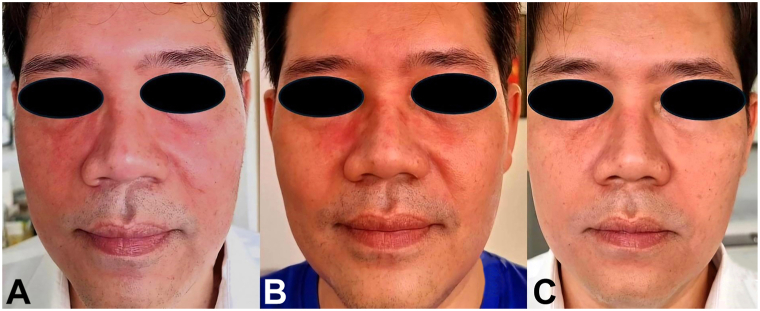
**A-C,** Facial *redness* with dry, scaly, hyperpigmented patches on the face, accompanied by itching and stinging sensations (**A**) An intense erythematous patch with peeling scales on his eyebrows and paranasal area (**B**) All erythematous patches, including dryness and roughness on both cheeks have been completely resolved, resulting in soft and smooth skin (**C**).

A 51-year-old man presented with facial redness and dry, scaly, hyperpigmented patches, accompanied by itching and stinging sensations on his face (Fig 1, *A*). He is in good health, without any chronic illnesses, and not taking any medications.

Three months ago, he noticed dryness on his face, which did not improve with moisturizing cream. He also experienced occasional itching and burning sensations. Later, he observed peeling scales around his eyebrows and the paranasal area. His cheeks became progressively drier and rougher, causing intense itchiness and a stinging sensation.

Physical examination revealed a mild erythematous patch with dry sandpaper-like roughness on both cheeks and hyperpigmented patches on both cheeks and forehead. The SSSB discovered 74 *Demodex* mites cm^2^ on the right cheek and 82 mites cm^2^ on the left cheek.

Based on these findings, he was diagnosed with demodicosis and treated with 2 doses of oral ivermectin (200 μg/kg, 1 week apart). Despite receiving treatment, his rash continued to worsen. He noticed that his cheeks looked redder than usual and were slightly swollen. Additionally, he reported experiencing increased dryness and stinging on his face. Furthermore, the rash on his eyebrows and the paranasal area had worsened and started to peel more, resembling seborrheic dermatitis (Fig 1, *B*). The SSSB demonstrated numerous *Demodex* mites, with 41 mites/cm^2^ on the right cheek and 59 mites/cm^2^ on the left cheek.

He continued taking oral ivermectin at the same dosage for an additional 4 weeks, and his symptoms gradually improved significantly. However, his skin still exhibited some dryness and roughness. Following this, he underwent oral ivermectin treatment for another 2 weeks. All clinical symptoms were entirely resolved, and the skin returned to its normal state of softness and smoothness (Fig 1, *C*).

Nonetheless, the SSSB still detected 9 mites/cm^2^ on the right cheek and 12 mites/cm^2^ on the left cheek.

## Discussion

Ivermectin is an antiparasitic drug that is effective against a wide range of parasites. It is quickly absorbed and metabolized in the liver, reaching its highest level in the bloodstream after 5 hours. The drug stays in the body for 36 hours and is mostly eliminated through feces (98%) and urine (1%).^6^^,^^7^

Ivermectin works by blocking the chemical transmission across nerve synapses that use Gamma-aminobutyric acid gated channels, leading to the paralysis and death of parasites. It is safe for humans because its targets are only found in the central nervous system, which ivermectin cannot reach due to the blood-brain barrier.^2^

There are no established dosages of ivermectin for treating demodicosis. The reported dosage is a single oral dose of 200 μg/kg. However, in some cases, the regimen may include repeated doses every 1 or 2 weeks for 2-5 times.^4^^,^^8^ This is because ivermectin has no ovicidal effect, and its plasma half-life is only 36 hours,^6^ while the larvae of *Demodex* mites hatch from the egg in 3-4 days and become adults in about 7 days.^2^

This patient was diagnosed with demodicosis and treated with oral ivermectin for 2 weeks. However, the condition worsened, leading to increased redness, swelling, dry, itchy, and peeling skin. Despite this, the patient continued taking oral ivermectin because the new rash had progressed from the previous rash, and the SSSB still discovered numerous mites. His symptoms began to improve significantly after the fourth week of treatment.

This phenomenon could be explained as a reaction caused by the rapid death of numerous *Demodex* mites due to oral ivermectin. The die-off symptom was first described by Jarisch-Herxheimer, who noted that patients undergoing penicillin treatment for syphilis experienced worsening symptoms before improving. This reaction can also be observed in candidiasis after antifungal treatment, known as the Candida die-off reaction.^9^

After a mite dies, its contents and the numerous bacteria it carries spread onto the skin.^2^^,^^10^ When many mites are eliminated, more waste is released onto the skin, triggering inflammatory responses in the patient. This can cause symptoms to worsen before gradually relieving and clearing up completely.

It is important to note that the patient's condition improved clinically after taking 4 to 8 doses of oral ivermectin. Therefore, even without initial signs of improvement, it is crucial to continue treatment if a die-off reaction of *Demodex* mites is suspected. Ivermectin cannot kill mite eggs, and the *Demodex* mite's life cycle is approximately 14 days. This might explain why patients with a high mite density may require treatment for more than 2-4 life cycles to see a decrease in mite density and an improvement in clinical symptoms, as demonstrated in this case.

The die-off reaction of *Demodex* mites following treatment with oral ivermectin may occur, especially in patients with a high mite density. Symptoms may worsen during the initial weeks before starting to improve with continued treatment. This condition is typically temporary. Symptoms improve as the mites are eliminated, eventually leading to symptom remission. Dermatologists should recognize and understand this condition to properly care for their patients.

## Conflicts of interest

None disclosed.

## References

[1] Forton F., Germaux M.A., Brasseur T. (2005). Demodicosis and rosacea: epidemiology and significance in daily dermatologic practice. J Am Acad Dermatol.

[2] Paichitrojjana A. (2022). Demodex: the worst enemies are the ones that used to be friends. Dermatol Reports.

[3] Salem D.A., El-Shazly A., Nabih N., El-Bayoumy Y., Saleh S. (2013). Evaluation of the efficacy of oral ivermectin in comparison with ivermectin-metronidazole combined therapy in the treatment of ocular and skin lesions of Demodex folliculorum. Int J Infect Dis.

[4] Truchuelo-Díez M.T., Alcántara J., Carrillo R., Martín M., Olasolo P.J., Pérez-Molina J. (2011). Demodicosis successfully treated with repeated doses of oral ivermectin and permethrin cream. Eur J Dermatol.

[5] Noguera-Morel L., Gerlero P., Torrelo A., Hernández-Martín Á. (2017). Ivermectin therapy for papulopustular rosacea and periorificial dermatitis in children: a series of 15 cases. J Am Acad Dermatol.

[6] Baraka O.Z., Mahmoud B.M., Marschke C.K., Geary T.G., Homeida M.M., Williams J.F. (1996). Ivermectin distribution in the plasma and tissues of patients infected with Onchocerca volvulus. Eur J Clin Pharmacol.

[7] Edwards G., Dingsdale A., Helsby N., Orme M.L., Breckenridge A.M. (1988). The relative systemic availability of ivermectin after administration as capsule, tablet, and oral solution. Eur J Clin Pharmacol.

[8] Dourmishev A.L., Dourmishev L.A., Schwartz R.A. (2005). Ivermectin: pharmacology and application in dermatology. Int J Dermatol.

[9] Jaiswal N., Kumar A. (2024). Candida die-off: adverse effect and neutralization with phytotherapy approaches. Toxicon.

[10] Lacey N., Delaney S., Kavanagh K., Powell F.C. (2007). Mite-related bacterial antigens stimulate inflammatory cells in rosacea. Br J Dermatol.

